# Exploring a Metacognitive Scaffolding-Based GenAI-Assisted Peer Feedback Provision Approach to Enhance Feedback Engagement Among Nursing Students

**DOI:** 10.3390/nursrep16060182

**Published:** 2026-05-27

**Authors:** Shuling Wei, Wei Wei

**Affiliations:** 1 Faculty of Applied Sciences, Macao Polytechnic University, Room M505, Meng Tak Building, Rua de Luis Gonzaga Gomes, Macao 999078, China; p2314941@mpu.edu.mo; 2 Center for Research Management and Services, Guangxi Open University, Room 1509, Distance Education Building, Nanning 530022, China

**Keywords:** GenAI, metacognitive scaffolding, peer feedback, engagement, nursing education

## Abstract

**Background:** Providing effective peer feedback is a challenge in nursing education. While Generative AI (GenAI) can assist, students often struggle with the task. Metacognitive scaffolding may help guide students through this complex process. **Aim:** This study aimed to evaluate the effects of a metacognitive scaffolding-based GenAI-assisted peer feedback provision (MGPFP) approach on nursing students’ feedback engagement and behavioral patterns. **Methods:** A quasi-experimental study was conducted with 71 nursing students. The experimental group (*n* = 35) used the MGPFP approach, while the control group (*n* = 36) used a standard GenAI-assisted approach without scaffolding. A Mann–Whitney U test was used to compare feedback engagement. Lag sequential analysis was used to examine feedback giving behavior patterns based on coded video data. **Results:** The experimental group reported significantly higher engagement than the control group across four dimensions: behavioral, cognitive, social, and emotional engagement. The experimental group generated 5219 coded behaviors, while the control group generated 1861. In the experimental group, common behaviors included referring external resources (19.58%), comparing and making judgements (17.80%), and recognizing the purpose (15.77%). Non-feedback behaviors were much higher in the control group (2.69%). Lag sequential analysis identified 17 significant sequences in the experimental group and 14 in the control group. **Conclusions:** Integrating metacognitive scaffolding into GenAI-assisted peer feedback can improve nursing students’ engagement and promote more productive and structured feedback behaviors. This approach is a valuable strategy for enhancing the quality of peer feedback in nursing education.

## 1. Introduction

Feedback is widely regarded as an important process for supporting learning and improving clinical competence in nursing education [1,2]. In nursing education, peer feedback is a well-established instructional approach that supports the development of nursing students ‘skills and knowledge [3,4]. Many nursing students also report positive attitudes toward peer feedback. This may be because peer feedback often involves less pressure than teacher feedback and allows students to prepare more effectively for future assessments [5]. However, some students show low engagement or complete feedback tasks in a superficial manner [6]. Others find it difficult to offer in-depth and meaningful comments due to limited domain knowledge, emotional barriers, or low confidence [7,8]. Despite these findings, existing studies largely rely on self-report measures and offer limited process-based evidence [7], which constrains understanding of how feedback engagement develops over time [9]. As a result, peer feedback in nursing education is often vague, lacks explanation, and provides few actionable suggestions [8,10,11]. Therefore, a clearer understanding of feedback engagement and behavioral patterns is needed to improve the quality and effectiveness of peer feedback in nursing education.

Interestingly, giving peer feedback was reported to be more beneficial than receiving feedback [11,12,13]. Peer feedback provision refers to how students identify problems, explain underlying reasons, and offer feasible suggestions [14]. Feedback provision behaviors are shaped by individual factors, including writing ability, motivation, and self-efficacy [15], as well as contextual factors, such as peer relations, task demands, and text quality [16]. However, empirical research on nursing students’ peer feedback provision remains limited, especially in GenAI-supported contexts [17,18]. Moreover, little is known about how peer feedback provision is generated in GenAI-supported environments or whether instructional support can foster more effective internal comparison.

GenAI is rapidly emerging as a new source of feedback and may help address several challenges in peer feedback implementation [19,20]. For example, GenAI has been shown to support nursing students’ critical thinking and problem-solving skills and to provide clearer and more actionable feedback [21,22]. GenAI may also reduce participation-related anxiety during peer interaction for some students [23]. In addition, GenAI-assisted peer feedback can offer instructional support during feedback provision and has been linked to improved feedback quality and learning gains [24,25,26]. Recent evidence in medical education shows that using GenAI to assist peer feedback can improve the specificity and actionability of students’ comments and is generally well received by learners [27].

However, the use of GenAI also has clear limitations. Unlike teachers or peers, GenAI cannot actively clarify misunderstandings when students provide weak or inaccurate prompts, which requires learners to judge prompt quality and revise it repeatedly to obtain effective feedback [28]. Although GenAI may reduce interaction-related anxiety for some students [23], its use also raises concerns about trust, ethics, and academic integrity [29]. More importantly, while peer feedback ideally engages learners in deep analysis and reflection, GenAI-assisted feedback may lead students to over-rely on AI for drafting comments. This could reduce the act of providing feedback to a more passive process, potentially diminishing critical thinking and metacognitive engagement [30,31]. For these reasons, there is a clear need to support sustained engagement in GenAI-assisted peer feedback rather than a replacement for students’ own judgment [20,27].

To address these mentioned limitations, metacognitive scaffolding has been proposed as an effective instructional strategy in GenAI-assisted learning. It deepens cognitive processing and enhances academic performance [32]. Several studies have introduced structured supports during feedback provision, such as guidance [33], prompts [34], and rubric-based prompts [35]. Metacognition is the ability to monitor, regulate, and reflect on one’s own cognitive processes [32]. Metacognitive scaffolding provides prompts and strategies that guide learners to plan, monitor, and evaluate their work when completing complex tasks [36]. Empirical studies have shown that metacognitive scaffolding can support higher-order thinking, improve problem-solving accuracy, and reduce cognitive load, with particularly strong effects for novice learners [37]. Learners who actively use scaffolds tend to show more expert-like learning behaviors, whereas those who use them less are more likely to struggle [26]. These findings suggest that scaffolding can enhance learning engagement and promote deeper understanding. However, although planning, monitoring, and evaluation are widely recognized as core metacognitive processes [38,39], there is still limited evidence on how metacognitive scaffolding influences peer feedback provision. This issue becomes more complex in learning environments that already include implicit scaffolds, such as exemplars, rubrics, and teacher comments [40], where the added value of structured metacognitive scaffolding remains unclear. In addition, prior studies indicate that nursing students’ feedback behaviors vary across instructional contexts, and that behavioral pattern analysis can be used to evaluate instructional effectiveness and guide intervention design [41,42]. Yet, in GenAI-assisted peer feedback settings, how students regulate their feedback behaviors and whether metacognitive scaffolding can shape these behaviors have not been systematically examined [17,18]. Therefore, this study aims to investigate the effects of having metacognitive scaffolding on nursing students’ feedback engagement with and behavior patterns in GenAI-assisted peer feedback giving tasks. Two research questions are addressed:

RQ1: To what extent does metacognitive scaffolding enhance engagement with GenAI when nursing students co-author peer feedback in a GenAI-assisted peer feedback environment?

RQ2: How does metacognitive scaffolding affect nursing students’ behavioral patterns during GenAI-assisted peer feedback co-authoring?

## 2. Methods

### 2.1. Participants and Context

This study adopted a quasi-experimental design. Participants were eligible if they had no prior training related to the intervention and had completed the required course Fundamentals of Nursing. This blended learning program has been implemented for more than 10 years. Most participants worked full-time and balanced work, family, and study responsibilities. Compared with students in traditional universities, they showed more diverse educational backgrounds and learning experiences, while ongoing work-study and family-study conflicts often limited their available study time and sustained engagement. A total of 71 nursing students aged 21–26 years (M = 22.42) were voluntarily recruited from a public open university in southern China. Students were assigned by class based on their online course registration an experimental group (*n* = 35; 8 males, 27 females) or a control group (*n* = 36; 8 males, 28 females). The experimental group used the metacognitive scaffolding-based GenAI-assisted peer feedback provision (MGPFP) approach, while the control group used conventional GenAI-assisted peer feedback provision (CGPFP) approach without scaffolding. No significant differences were found in age or gender between groups (*p* > 0.05). Both groups were taught by the same instructor with over 10 years of teaching experience, who completed two 8 h training workshops on GenAI-supported teaching. All task sessions were video-recorded and evaluated by the lead researcher. Informed consent was obtained from all participants.

### 2.2. Data Collection

#### 2.2.1. Feedback Engagement Scale

Nursing students’ engagement with GenAI-drafted feedback during their co-authoring of peer feedback was measured using the 23-item, four-dimensional scale developed by Gan et al. [43], which assesses behavioral, cognitive, social, and emotional dimensions. All items were rated on a 5-point Likert scale ranging from 1 (“strongly disagree”) to 5 (“strongly agree”). Higher scores indicated greater reported engagement in integrating GenAI feedback into the final feedback provided to peers. In this study, the scale showed high internal consistency, with Cronbach’s alpha coefficients ranging from 0.83 to 0.92 across the four dimensions, demonstrating strong reliability.

#### 2.2.2. The Coding Scheme for Nursing Students’ GenAI-Assisted Peer Feedback Provision Behaviors

To capture nursing students’ GenAI-assisted peer feedback provision behaviors, a coding scheme was developed in several steps. First, an initial coding framework was synthesized from prior literature [18,40,44]. The framework and sample task videos were then reviewed on a small-scale subset from both groups to ensure all relevant behaviors were captured. Next, a panel of two subject matter experts evaluated the scheme’s suitability. The coding framework was refined based on their feedback (Table 1). Two trained coders analyzed video recordings and post-experiment interviews. The videos captured all observable behaviors in co-author peer feedback with GenAI chatbot with or without metacognitive scaffolding. For example, recognizing the purpose was coded when a student explained why feedback was important and consulted a GenAI chatbot to clarify the task purpose. The inter-rater reliability reached a Cohen’s Kappa of 0.82, which indicates acceptable agreement [45]. Any disagreements were discussed until consensus was reached.

### 2.3. Development of Metacognitive Scaffolding-Based GenAI-Assisted Peer Feedback Provision (MGPFP) Approach

#### 2.3.1. The MGPFP Environment

**Figure 1:**
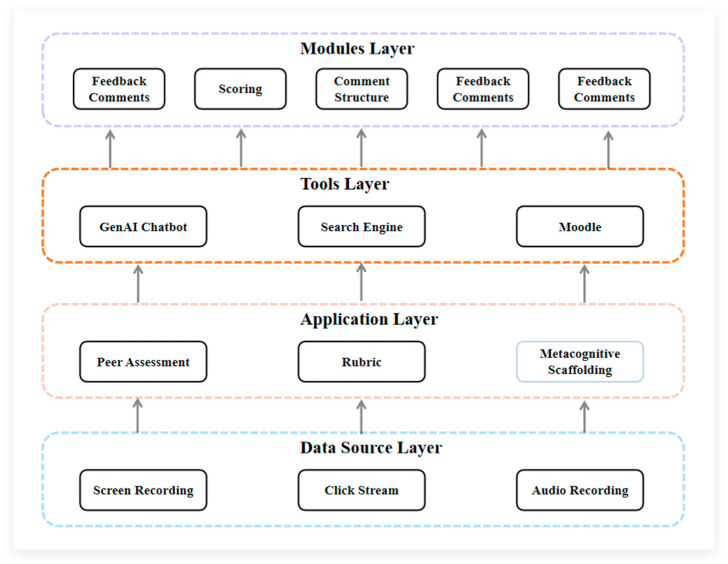
Metacognitive scaffolding-based GenAI-assisted peer feedback provision environment.

This study implemented a peer feedback provision task in the Moodle (Figure 1). A GenAI chatbot fine-tuned from DeepSeek-R1 was used to support the feedback process (See Supplementary Materials for fine-tuning details and the complete system prompt). DeepSeek-R1 is an open-source large language model that has shown strong performance in structured reasoning tasks, including text-based case analysis and clinical reasoning [46]. Moodle integrated key learning resources, including instructional materials, exemplars, rubrics, and peer’s work, which students could access when needed [40,47,48,49]. In this study, the experimental group co-constructing peer feedback with GenAI chatbot with metacognitive scaffolding (MGPFP), while the control group collaborated with GenAI-assisted peer feedback without scaffolding.

#### 2.3.2. The Clinical Reasoning Training with the MGPFP Approach

**Figure 2:**
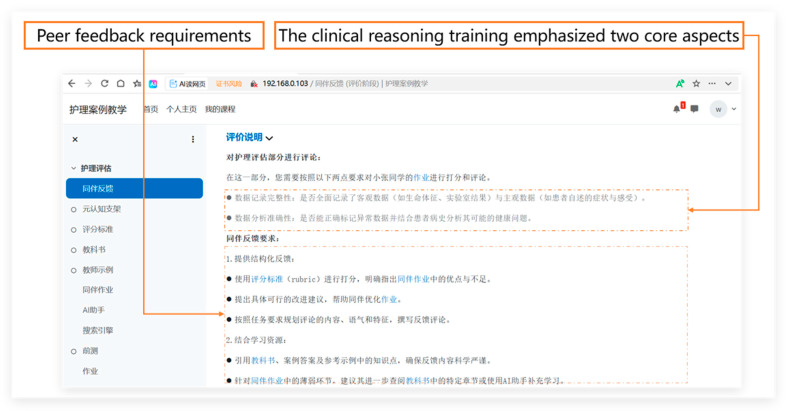
The clinical reasoning training with the MGPFP approach in Moodle (screenshot). The left navigation menu includes: Home, Personal Page, My Courses, Peer Feedback, Metacognitive Scaffolding, Rubric, Textbook, Teacher Example, Peer Work, AI Assistant, Search Engine, Pre-test, Assignment. The right panel shows the peer feedback task: a clinical reasoning case requiring comments on a peer’s nursing assessment.

The training focused on two core aspects (Figure 2): (a) whether peer work fully presented both objective and subjective clinical data, and (b) whether abnormal data were analyzed in relation to patient history. When uncertainty occurred, students in the experimental group could access the “metacognitive scaffolding” module in Moodle for immediate guidance, whereas students in the control group organized the feedback process independently. This design aimed to support the quality of GenAI-assisted peer feedback through metacognitive scaffolding.

**Figure 3:**
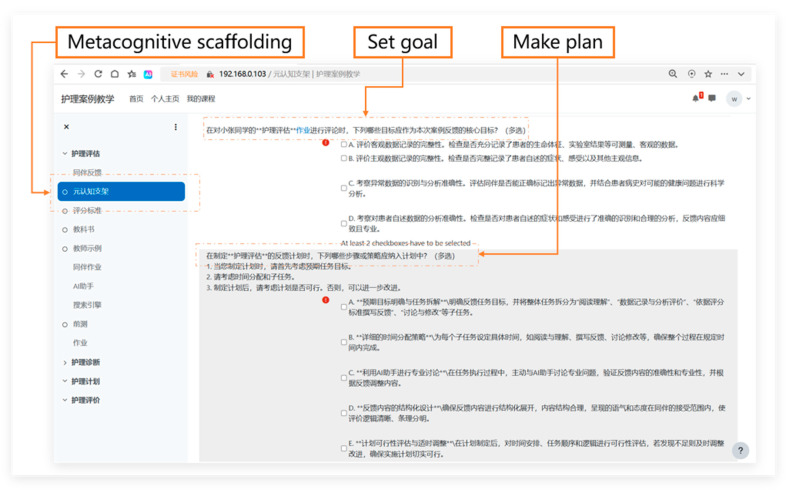
Metacognitive scaffolding (screenshot). The right panel presents metacognitive scaffolding questions. First, students set feedback goals (e.g., completeness of objective/subjective data, accuracy of abnormal data identification). Second, they make planning strategies (e.g., task breakdown, time allocation, AI discussion, structured design, feasibility evaluation).

Metacognitive scaffolding was embedded in Moodle as multiple-choice prompts, with each option linked to key metacognitive strategy dimensions [50]. When students were unsure about strategy use, they could access the scaffolding module via the Moodle navigation bar (Figure 3). Planning scaffolding guided students to select core feedback goals, such as the completeness of objective data or the accuracy of abnormal data analysis. For instance, the options included “Evaluate completeness of objective data documentation” or “Evaluate completeness of subjective data documentation.” Monitoring scaffolding was triggered by teacher reminders during task execution or after five minutes of inactivity, prompting self-checks of clinical logic consistency and constructive tone. For example, the options included “Confirm clear labelling of all data omissions” or “Verify integration of abnormal signs with patient history.” Evaluation scaffolding supported students in identifying sources of difficulty, reflecting on strategy effectiveness, and confirming whether their feedback met professional standards. For instance, the options included “Insufficient feedback comprehensiveness” or “Unprofessional or inconsistent feedback tone.” The control group received no scaffolding prompts and relied on prior experience to complete peer feedback.

#### 2.3.3. The Feedback Process of the MGPFP Approach

**Figure 4:**
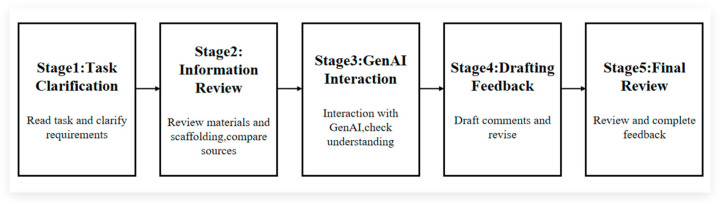
The feedback process of the MGPFP approach.

In this study, a metacognitive scaffolding-based GenAI-assisted peer feedback provision (MGPFP) approach was developed based on representative prior studies [18,40,51]. During the peer feedback provision task, students were required to actively use Moodle resources, GenAI chatbot, and search engines to integrate prior and new knowledge when providing feedback to peers. The approach consists of five sequential stages. First, students read the peer feedback provision task and clarify task requirements. Second, they review supporting materials or metacognitive scaffolding and compare multiple sources of information to identify key issues for feedback. Third, students interact with GenAI chatbot to obtain additional information, check their understanding, and refine comparisons. Fourth, they draft feedback comments and revise them based on the results of these comparisons. Finally, students complete the peer feedback provision task by reviewing and refining their feedback comments. The instructional process of the MGPFP approach is illustrated in Figure 4.

### 2.4. Experimental Procedure

**Figure 5:**
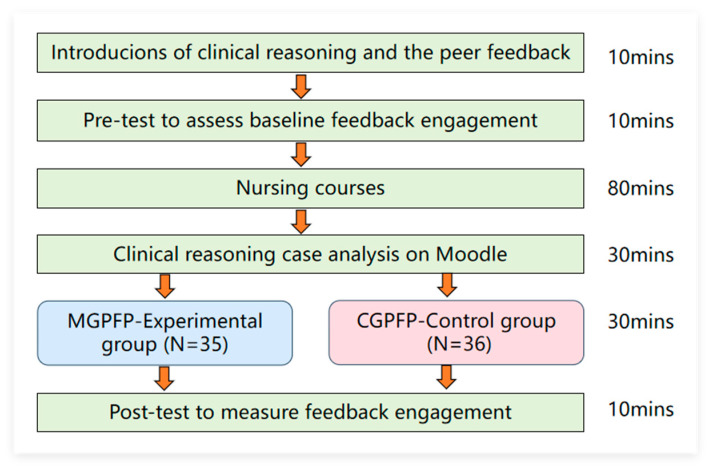
The experimental procedure.

The experimental procedure is shown in Figure 5. At the start, the course instructor introduced the learning goals for clinical reasoning and the peer feedback provision task. A 10 min pre-test was then administered to assess students’ baseline feedback engagement. Next, students attended two weeks of foundational nursing courses (80 min per week). After that, they completed a 30 min clinical reasoning case analysis on the Moodle platform. Each student then spent 30 min providing peer feedback on two classmates’ case analyses. The experimental group used metacognitive scaffolding-based GenAI-assisted peer feedback provision (MGPFP) approach, in which scaffolding was embedded in the feedback process. The control group used conventional GenAI-assisted peer feedback provision (CGPFP) without scaffolding. After the intervention, both groups completed a post-test to measure feedback engagement.

### 2.5. Data Analysis

To address RQ1, a Mann–Whitney *U* test was used to compare feedback engagement level between the experimental and control groups when co-constructing peer feedback with GenAI chatbot with and without metacognitive scaffolding. This nonparametric test was selected because the sample size was small (*n* < 100) and the data may not follow a normal distribution. Feedback engagement was examined as it reflects key indicators of peer feedback effectiveness, particularly when GenAI may influence both the content and depth of student interactions [34]. The significance level was set at *p* < 0.05. All analyses were conducted using SPSS 27.0.

To address RQ2, students’ behaviors in drafting peer feedback with GenAI in control and experimental groups during case analysis on the Moodle platform were video-recorded. Using the coding scheme and videos, two researchers coded students’ peer feedback drafting behaviors, recording each observed action [52]. In this study, an “observed action” was defined as a single behavioral event. Onset was marked by a student browsing a page or typing; offset was marked by switching pages or submitting a response. The interaction log was segmented using semantic and task transition rules: a new action was coded when (a) the student moved to a new page or opened learning resources (e.g., instructional materials, exemplars, rubrics, peer work), (b) the student read or revised new AI-generated content, or (c) a clear pause (>5 s) occurred followed by a shift in content. To examine sequential behavior patterns, lag sequential analysis (LSA) was employed. LSA identifies behavior transitions that occur more frequently than expected by chance, revealing temporal learning patterns [53]. Behavioral sequence analysis has been applied to interpret how students engage cognitively and socially in learning tasks [54]. Using GSEQ 5.1.23 software, we applied LSA to the students’ feedback provision behaviors. Z-scores above 1.96 indicated significant associations. Behavior transition diagrams were then created to compare patterns between the experimental and control groups.

## 3. Results

### 3.1. Analysis of Feedback Engagement

To ensure baseline equivalence, pretest Mann–Whitney U tests indicated no significant differences between the experimental and control groups in behavioral engagement (U = 617.50, *p* = 0.885, r = 0.02), cognitive engagement (U = 614.50, *p* = 0.857, r = 0.02), social engagement (U = 586.00, *p* = 0.609, r = 0.06), or emotional engagement (U = 590.00, *p* = 0.640, r = 0.06), supporting group equivalence before the intervention. Posttest results (Table 2) revealed that the experimental group scored significantly higher than the control group across all feedback engagement dimensions. Behavioral (U = 270.00, *p* < 0.001, r = 0.49), cognitive (U = 282.00, *p* < 0.001, r = 0.48), and social engagement (U = 307.00, *p* < 0.001, r = 0.45) showed large effect sizes, whereas emotional engagement (U = 407.00, *p* = 0.009, r = 0.31) showed a moderate effect. Median and interquartile ranges confirmed these patterns, with the experimental group consistently demonstrating higher central tendency and minimal overlap with the control group. These results indicate that embedding metacognitive scaffolding in GenAI-assisted peer feedback provision significantly enhanced students’ overall and multidimensional feedback engagement when co-constructing peer feedback with GenAI compared with their colleagues in the control group without metacognitive scaffolding.

### 3.2. Analysis of Nursing Students’ GenAI-Assisted Peer Feedback Provision Behavioral Patterns

During the peer feedback provision task, all student actions were automatically logged by Moodle, and screen recordings captured on-screen activities. Sequential analysis was then conducted using the Generalized Sequential Querier (GSEQ) to examine behavioral patterns in the experimental and control groups. In total, 35 students in the experimental group generated 5219 coded behaviors, while 36 students in the control group generated 1861 coded behaviors.

**Figure 6:**
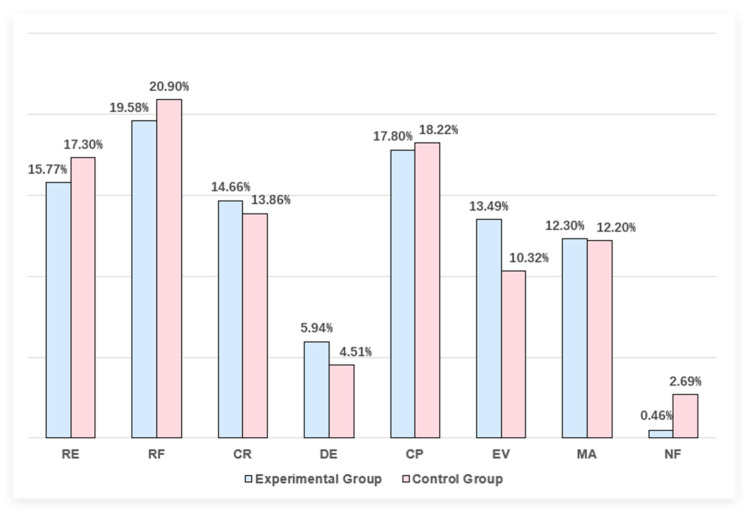
Percentages of coded behaviors in the two groups.

Figure 6 shows the frequency and proportion of coded feedback drafting behaviors in both groups. In the experimental group, the most frequent behaviors were referring external resources (RF, 19.58%), comparing and making judgements (CP, 17.80%), and recognizing the purpose of providing feedback (RE, 15.77%). In the control group, higher proportions were found for recognizing the purpose (RE,17.30%), referring external resources (RF,20.90%), comparing and making judgements (CP,18.22%), and non-feedback behaviors (NF, 2.69%). Notably, NF behaviors in the control group were more than six times those in the experimental group.

Based on the adjusted residuals in Table 3 and Table 4, 17 significant sequences were identified in the experimental group and 14 in the control group.

**Figure 7:**
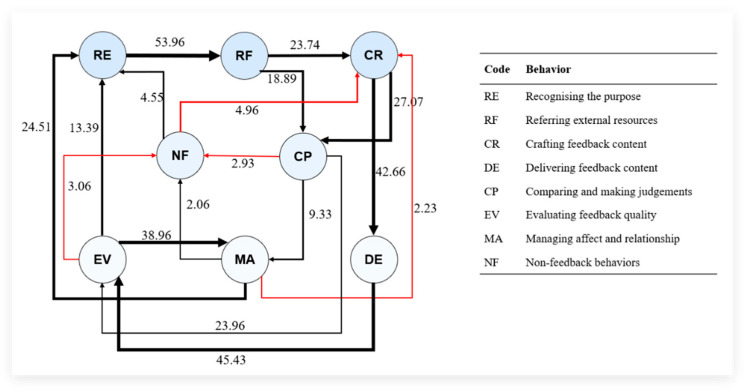
The transition diagram of significant behavioral sequences of the experimental group.

**Figure 8:**
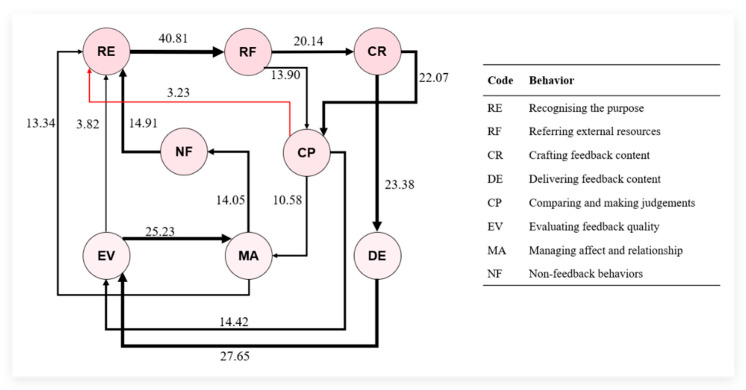
The transition diagram of significant behavioral sequences of the control group.

Figure 7 and Figure 8 present the transition diagrams of significant behavioral sequences for the two groups. Black lines indicate sequences shared by both groups, while red lines represent sequences unique to either the experimental or the control group. In both groups, the most prominent pathway was from recognizing the purpose (RE) to referring external resources (RF). This finding shows that, regardless of condition, students typically began the peer feedback provision task by clarifying the task purpose and then consulting assessment criteria, external resources, or GenAI. However, the Z value for this transition was higher in the experimental group than in the control group (53.96 vs. 40.81). This finding indicates that students in the experimental group relied on criteria, external resources, or GenAI in a more stable way after clarifying feedback goals.

In the middle and later stages of the feedback provision task, the two groups showed clear differences in behavioral sequences. In the experimental group, the most prominent sequences were Crafting feedback content (CR) → Delivering feedback content (DE) (Z = 42.66) and DE → EV (Z = 45.43). These sequences formed a structured and coherent feedback pathway: RE → RF → CR → CP/DE → EV → MA → task continuation. This path represents a complete cycle from task understanding, criteria referencing, idea generation, comparative evaluation, to quality assessment. It also indicates that managing affect and relationship (MA) in the experimental group served as a regulatory step supporting feedback quality rather than an endpoint. In contrast, the control group’s feedback paths were more fragmented. Although the initial structure (RE → RF → CR) was similar, sequences often ended with MA → NF → RE. This pattern suggests that without scaffolding, MA becomes a weak point. When students feel uncertain about their emotional expression, they tend to reduce engagement and shift to task completion or non-feedback behaviors rather than continuing to monitor or refine feedback quality.

Red lines in Figure 7 show four unique sequences in the experimental group, where NF behaviors were followed by a return to CR, indicating self-checking after emotional management. The MA → CR path further confirms that students revisited content evaluation after completing emotional regulation. In Figure 8, some control group students repeatedly compared criteria, external resources, or GenAI chatbot (RF → CR → CP) before generating feedback, but often engaged in non-feedback behaviors afterward. Others skipped comparisons, moving directly from RF → CR → DE → EV, yet still showed NF behaviors. This pattern suggests that some students did not fully understand the feedback provision task or were less motivated to engage. Additionally, red lines in Figure 8 indicate that some students returned to RE after CP, showing incomplete comprehension of the feedback requirements. Overall, these results suggest that completing peer feedback without scaffolding is more challenging and prone to fragmented or disengaged behaviors.

## 4. Discussion

### 4.1. Feedback Engagement

In response to RQ1, the results show that students in the experimental group demonstrated higher behavioral, cognitive, social, and emotional engagement in co-constructing peer feedback with GenAI chatbot than those in the control group. In other words, directly coping and pasting GenAI feedback into their peer feedback are less likely to happen in the experimental group. From a metacognitive perspective, the metacognitive scaffolding served as structured support that made the whole peer feedback giving process more cognitive demanding, including making plan, monitoring, and evaluating GenAI output. This support helped students gradually internalize these strategies and engage in feedback activities in a more expert-like manner. Prior studies in digital learning contexts report similar effects, showing that metacognitive scaffolding improves monitoring accuracy and problem-solving efficiency [37,55], and promotes deeper cognitive engagement and self-regulation [26]. In addition, during peer assessment, students generate internal feedback by comparing their work with that of peers. This process strengthens metacognitive reflection and self-evaluation [40,56]. Together, internal feedback and external peer feedback support sustained engagement. Therefore, integrating metacognitive scaffolding into peer feedback helps students manage feedback engagement.

### 4.2. Nursing Students’ Feedback Behavior Patterns

In response to RQ2, the behavioral sequence analysis shows clear differences between the experimental and control groups, indicating that metacognitive scaffolding led to meaningful changes in nursing students’ behaviors in integrating GenAI feedback into their final feedback to their peers. These differences can be explained from the perspectives of metacognitive strategies, GenAI digital affordances, scaffolding support, and emotional regulation during peer feedback.

First, the experimental group demonstrated structured, coherent, and strategy-driven behavioral pathways. These pathways reflect the key role of metacognitive scaffolding in supporting intentional, controlled, and reflective feedback giving activities. By making planning, monitoring, and evaluation processes explicit, the scaffolding helped students initiate and sustain core stages of feedback generation. The observed sequences align with established metacognitive models, suggesting that students gradually internalized a complete feedback cycle rather than engaging in fragmented or surface-level actions [57,58]. In contrast, the control group followed more linear and shallow sequences. Although they completed basic evaluation steps (RE → RF → CR → DE), they showed no stable transitions toward evaluating feedback quality (EV), indicating limited self-review of feedback quality. This finding is consistent with prior research showing that, without explicit scaffolding, nursing students tend to focus on task completion and surface correctness rather than higher-order judgment [55].

Second, the digital affordances of GenAI further explain the increase in higher-level behaviors in the experimental group. GenAI provided immediate examples, explanations, alternative expressions, and structured reasoning prompts, which reduced cognitive load and supported decision-making and reflection [37]. Significant transitions such as CR → CP and DE → EV indicate that students were able to interpret, organize, and revise their own feedback to other peers more effectively with GenAI support. These patterns suggest that GenAI, when guided by scaffolding, facilitates deeper engagement with feedback content rather than passive reliance on GenAI output.

Third, differences in emotional regulation behaviors highlight the importance of metacognitive scaffolding in feedback processes. From a metacognitive perspective, managing affect (MA) is a key regulatory component. In the experimental group, MA was integrated into monitoring processes, allowing students to adjust tone and then return to feedback goals and quality reflection. In contrast, control group students often showed uncertainty during MA, which led to reduced engagement or shifts to non-feedback behaviors. This pattern reflects insufficient regulatory support and aligns with research on metacognitive laziness in technology-supported learning [31].

Finally, the marked difference in non-feedback (NF) behaviors further supports the combined effect of scaffolding and GenAI. NF behaviors were rare in the experimental group, indicating sustained focus and confidence. In the control group, NF behaviors appeared more frequently and were linked to emotional pressure and feedback difficulty, suggesting lower self-efficacy. This finding echoes studies showing that lack of scaffolding increases avoidance behaviors and reduces persistence in peer feedback provision tasks [59,60]. For students with low self-efficacy and weak digital literacy, teachers should simplify prompts, increase modeling and step-by-step guidance, and provide human support. This can reduce cognitive load and gradually build students’ confidence in using the approach.

## 5. Conclusions

This study developed and tested a metacognitive scaffolding peer feedback provision approach in a GenAI-assisted learning environment. The evidence showed that the MGPFP approach showed promising potential to enhance students’ peer feedback engagement. Integrating metacognitive scaffolding also promoted metacognitive behaviors, addressing common challenges in GenAI-assisted learning. Instructors can guide students to organize knowledge, monitor progress, and reflect on peer feedback, supporting clinical reasoning competency in nursing education.

Several limitations should be noted. The sample was small and drawn from a single school, which may limit generalizability. Only feedback engagement was measured; other learner characteristics, such as achievement or feedback literacy, were not assessed. The intervention was short-term, and long-term effects on clinical reasoning or sustained motivation remain unknown. Although behavioral sequences were analyzed using GSEQ, deeper investigation of learners’ metacognitive processes during peer feedback is warranted.

Future studies could use larger and more diverse samples to improve generalizability. We measured only immediate post-intervention effects. Therefore, it is still unclear whether the observed metacognitive behaviors last over time or apply to real clinical settings. This question should be addressed in future longitudinal studies. Combining behavioral data with rich qualitative methods, such as think-aloud protocols or epistemic network analysis (ENA), can provide deeper insights into students’ reasoning and metacognitive processes. To support the transfer of metacognitive behaviors to real-world clinical practice, future studies could use the following strategies: (a) include metacognitive scaffolding across multiple clinical courses or simulation sessions to encourage habit formation; (b) set up peer mentoring, where trained students guide untrained peers.

## Figures and Tables

**Table 1:** The coding scheme for nursing students’ GenAI-assisted peer feedback provision behaviors.

Code	Behavior	Descriptions
RE	Recognizing the purpose	A student recognizes why feedback matters and may consult GenAI chatbot to clarify task purposes.
RF	Referring external resources	A student refers to rubrics, exemplars, textbooks, scaffolding, or GenAI chatbot to interpret evaluative standards.
CR	Crafting feedback content	A student plans comments, analyses issues, and may refine wording with GenAI support.
DE	Delivering feedback content	A student provides clear, actionable, and respectful comments, sometimes assisted by GenAI chatbot.
CP	Comparing and making judgements	A student compares peer work with criteria, external sources, or GenAI chatbot suggestions to justify judgments.
EV	Evaluating feedback quality	A student reviews feedback clarity and usefulness and may use GenAI chatbot to check coherence or tone.
MA	Managing affect and relationship	A student maintains an appropriate tone and may use GenAI chatbot to ensure empathy and courtesy.
NF	Non-feedback behaviors	A student engages in off-task actions, including irrelevant GenAI chat or copying GenAI-generated text.

**Table 2:** Mann–Whitney *U* test results for students’ feedback engagement between groups.

Variable	Item	EG	CG	Mean Rank (EG/CG)	Sum of Ranks (EG/CG)	*U*	Z	*p*	r
		M (SD)/Median [IQR]	M (SD)/Median [IQR]						
Feedback behavioral engagement	Pre	24.46 (3.34)/23.00 [5.00]	24.39 (3.45)/23.00 [4.75]	36.36/35.65	1272.50/1283.50	617.50	–0.15	0.885	0.02
	Post	28.66 (3.45)/28.00 [5.00]	25.00 (3.58)/24.00 [5.50]	46.29/26.00	1620.00/936.00	270.00	–4.17	<0.001	0.49
Feedback cognitive engagement	Pre	20.97 (2.55)/20.00 [4.00]	21.11 (2.76)/21.00 [4.00]	35.56/36.43	1244.50/1311.50	614.50	–0.18	0.857	0.02
	Post	24.29 (2.77)/24.00 [4.00]	21.39 (2.68)/21.00 [4.00]	45.94/26.33	1608.00/948.00	282.00	–4.03	<0.001	0.48
Feedback social engagement	Pre	21.31 (2.64)/21.00 [4.00]	20.97 (2.36)/21.00 [3.00]	37.26/34.78	1304.00/1252.00	586.00	–0.51	0.609	0.06
	Post	23.63 (2.66)/23.00 [4.00]	21.22 (2.42)/21.00 [2.75]	45.23/27.03	1583.00/973.00	307.00	–3.76	<0.001	0.45
Feedback emotional engagement	Pre	14.09 (2.15)/14.00 [4.00]	13.81 (1.92)/13.50 [3.00]	37.14/34.89	1300.00/1256.00	590.00	–0.47	0.640	0.06
	Post	15.46 (2.12)/16.00 [3.00]	14.06 (2.08)/14.00 [3.75]	42.37/29.81	1483.00/1073.00	407.00	–2.59	0.009	0.31

Note: EG = experimental group; CG = control group. M = mean; SD = standard deviation; IQR = inter-quartile range.

**Table 3:** The adjusted residuals tables for the experimental group.

Given:	RE	RF	CR	DE	CP	EV	MA	NF
RE	0	53.96	−14.37	−8.28	−16.53	−13.55	−12.78	−2.18
RF	−9.54	0	23.74	−9.5	18.89	−15.54	−14.65	−2.51
CR	−14.07	−16.48	0	42.66	27.07	−12.55	−11.83	−2.02
DE	−8.11	−9.5	−7.67	0	−8.82	45.43	−6.82	−1.17
CP	−4.83	−18.95	0.8	−8.82	0	23.96	9.33	2.93
EV	13.39	−15.25	−12.1	−7.1	−14.17	0	38.96	3.06
MA	24.51	1.43	2.23	−6.77	−13.5	−11.07	0	2.06
NF	4.55	−2.51	4.96	−1.17	−2.33	−1.91	−1.8	0

**Table 4:** The adjusted residuals tables for the control group.

Given:	RE	RF	CR	DE	CP	EV	MA	NF
RE	−9.60	40.81	−8.42	−4.56	−9.90	−7.12	−7.82	−3.49
RF	−3.81	−11.12	20.14	−4.82	13.90	−7.52	−8.27	−2.27
CR	−7.92	−8.90	−6.94	23.38	22.07	−5.87	−6.45	−2.88
DE	−4.29	−4.82	−3.76	−2.04	−4.43	27.65	−3.50	−1.56
CP	3.23	−10.47	−0.69	−4.43	−9.61	14.42	10.58	0.33
EV	3.82	−7.30	−4.79	−3.09	−6.70	−4.82	25.23	−2.36
MA	13.34	−2.46	1.48	−3.28	−7.12	−5.12	−5.63	14.05
NF	14.91	−2.98	−2.88	−1.56	−3.38	−2.43	−2.67	−1.19

## Data Availability

The data presented in this study are available on request from the corresponding author due to ethical and privacy restrictions imposed by the institutional review board (IRB), as the dataset contains potentially identifiable information from a small sample of nursing students. Requests for access should be sent to the corresponding author and will be reviewed in accordance with the IRB’s data sharing guidelines.

## Supplementary Materials

The following supporting information can be downloaded at: https://www.mdpi.com/article/10.3390/nursrep16060182/s1.

## Public Involvement Statement

There was no public involvement in any aspect of this research.

## Guidelines and Standards Statement

This manuscript was drafted against the checklist for reporting of studies of reliability and agreement (GRAAS) Statement: Guidelines for Reporting Reliability and Agreement Studies.
