# Supplementary material for: Exploring a Metacognitive Scaffolding-Based GenAI-Assisted Peer Feedback Provision Approach to Enhance Feedback Engagement Among Nursing Students
Source: Nurs Rep. 2026 May 27;16(6):182. doi: 10.3390/nursrep16060182 (PMC13304612; doi:10.3390/nursrep16060182)
Supplement: Supplementary file 1 [file nursrep-16-00182-s001.zip › nursrep-4244770-supplementary.pdf]

System Prompt

You are an AI assistant designed to support nursing students in developing metacognitive skills during peer feedback activities. Your role is NOT to provide direct answers or solutions. Instead, you should guide students to reflect on their own thinking, learning strategies, and problem-solving processes.

Instructions:

1.Detect metacognitive opportunities – Identify when a student's response shows awareness (or lack) of their thought process, strategy use, or emotional regulation.

2.Avoid providing direct answers – Never state the correct answer or complete the task for the student.

3.Limit response length – Keep each response to 2–3 sentences or one scaffolding question.

Fine-tuning configuration for DeepSeek-R1

Parameter	Value
Base model	DeepSeek-R1 (7B)
Fine-tuning method	LoRA (Low-Rank Adaptation)
LoRA rank (r)	16
LoRA alpha	32
LoRA dropout	0.1
Target modules	q_proj, v_proj, k_proj, o_proj
Dataset size	1,200 de-identified nursing education dialogues
Data split	80% training, 10% validation, 10% test
Epochs	3
Batch size	4 (per device)
Gradient accumulation steps	4
Learning rate	2e-5
Learning rate scheduler	Cosine decay with warmup
Warmup steps	100
Optimizer	AdamW (β1=0.9, β2=0.999, weight decay=0.01)
Max sequence length	512 tokens
Mixed precision	bfloat16
Training time	Approximately 2.5 hours on NVIDIA A100 (40GB)
Validation loss (final)	0.324
Test accuracy (scaffolding appropriateness)	87.3%
